# Unravelling the association between renal functions and anti thyroid peroxidase antibody levels in newly diagnosed cases of hypothyroidism in elderly

**DOI:** 10.3389/fendo.2025.1572675

**Published:** 2025-08-27

**Authors:** Gayathri Devi D, Kishore Kumar Behera, Manaswini Mangaraj, Gautom Kumar Saharia

**Affiliations:** ^1^ Department of Biochemistry, All India Institute of Medical Sciences (AIIMS), Bhubaneswar, Odisha, India; ^2^ Department of Endocrinology, All India Institute of Medical Sciences (AIIMS), Bhubaneswar Odisha, India

**Keywords:** hypothyroidism, elderly population, anti TPO antibodies, EGFR, thyroid function, renal function

## Abstract

**Introduction:**

Elderly population is more susceptible to both hypothyroidism and chronic kidney disease (CKD) independently and it is very crucial to assess kidney function in elderly hypothyroid patients in relation to autoimmunity. There are previous studies showing a correlation between eGFR and TSH, T3, and T4 in the elderly population but no such studies correlating the estimated GFR (eGFR) with the anti-thyroid antibodies of elderly hypothyroid patients are available. This study aimed to assess renal function in newly diagnosed elderly hypothyroid patients and correlate findings with anti-thyroid peroxidase (anti-TPO) levels.

**Methods:**

We conducted a cross-sectional study with 128 participants aged 60 years and above after obtaining ethics committee approval. All cases were newly diagnosed patients of hypothyroidism whether clinical or subclinical. The control group consisted of age and sex matched individuals with normal thyroid profile status. Fasting blood samples were collected and serum thyroid parameters, glycemic and renal markers were evaluated, and statistical analyses were performed.

**Results:**

Hypothyroid patients exhibited elevated anti-TPO levels and markers of renal dysfunction, including increased urea, uric acid, and urine albumin/creatinine ratio. Multiple regression analysis identified anti-TPO as an independent predictor of kidney function. Subgroup analysis revealed significantly lower eGFR and elevated renal markers in anti-TPO positive patients.

**Discussion:**

The interplay between thyroid dysfunction and renal impairment is particularly significant in the elderly, who are more vulnerable to both conditions. Elevated anti-TPO levels are associated with renal dysfunction in elderly hypothyroid patients, suggesting a potential role for anti-TPO in renal impairment.

## Graphical Abstract

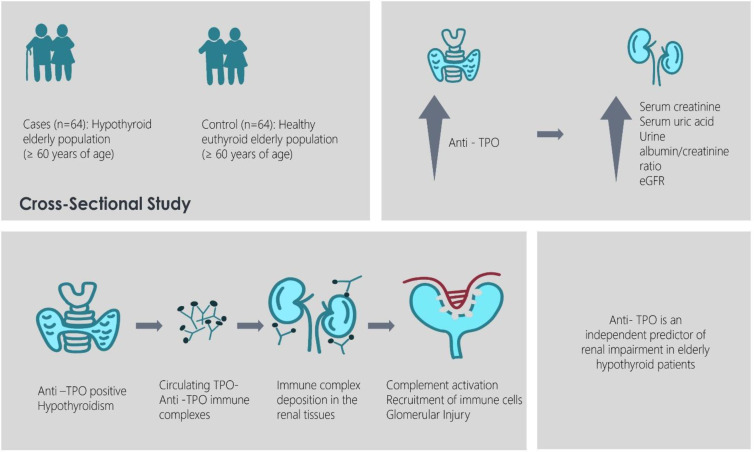

## Introduction

The thyroid hormone, beyond its metabolic role, contributes to the development and functioning of the kidney, influencing hemodynamic changes and the glomerular filtration rate (GFR). Both hypo and hyperfunction of the thyroid can alter kidney function (1). Autoimmune diseases such as SLE, Goodpasture syndrome, and Hashimoto’s thyroiditis also impact kidney function. While there is evidence of Thyroglobulin antibody affecting renal function, leading to conditions like glomerulonephritis and nephrotic syndrome, data on the impact of Anti Thyroid peroxidase antibody (Anti-TPO) on renal function is limited (2).

The elderly population experiences age-related fibrosis and atrophy in the thyroid gland, compromising its function. The incidence of thyroid nodules and thyroid tumours also increases with age. The prevalence of autoantibodies is higher with age, particularly in females. Given that this group is more susceptible to interrelated disorders and that both hypothyroidism and chronic kidney disease (CKD) independently increase cardiovascular disease risk, it is crucial to assess kidney function in elderly hypothyroid patients in relation to autoimmunity (3).

Autoimmunity in the thyroid gland is generally identified by the autoantibodies developed in thyroid gland, specifically anti-TPO and anti-Tg antibodies. Recent studies have demonstrated the value of autoantibodies as early diagnostic markers in various diseases such as rheumatoid arthritis, cancer, and celiac disease (4). Thyroid peroxidase (TPO) which is a glycosylated membrane-bound haemoprotein, plays a pivotal role in the biosynthesis of thyroid hormones. Anti-TPO antibodies are specific for the autoantigen TPO and they are the most common anti-thyroid autoantibody found in approximately 90% of Hashimoto’s thyroiditis, 75% of Graves’ disease, and 10-20% of nodular goitre or thyroid carcinoma cases. Interestingly, 10-15% of normal individuals can have high-level of anti-TPO antibody titres (5–7). We have also seen that ferritin concentrations are decreased in anti-TPO negative hypothyroidism, but in the case of anti-TPO positive hypothyroidism, the ferritin concentrations are raised. Hence, hypothyroidism should not always be considered an iron deficiency state (8).

Thyroid and kidney functions are closely interconnected—thyroid hormones are essential for kidney growth, development, and maintaining electrolyte balance, while the kidneys play a key role in metabolizing and eliminating these hormones. This bidirectional relationship makes it challenging to determine a clear cause-and-effect link between thyroid and kidney dysfunction. However, examining how frequently these conditions occur together may help identify populations at greater risk like the elderly group and improve early recognition and intervention strategies. While previous studies have shown a correlation between eGFR and TSH, T3, and T4 in the elderly population, our extensive literature search revealed no studies correlating the estimated GFR (eGFR) with the anti-thyroid antibodies of elderly hypothyroid patients.

Considering these findings, our study aims to evaluate renal function in newly diagnosed elderly hypothyroid patients. It further explores the correlation of eGFR between Anti TPO positive and negative elderly hypothyroid patients to provide new insights into the interplay between thyroid autoimmunity and renal function in this population.

## Methodology

Our study was a cross-sectional hospital-based study done in a single centre i.e. All India Institute of Medical Sciences (AIIMS) Bhubaneswar, which is a premier tertiary care centre in the eastern region of India. Patients of 60 years and above visiting the Endocrinology OPD who are advised to do thyroid Profile and anti TPO levels were recruited. The ethical clearance for the study was obtained from the Institutional Ethical Committee of AIIMS Bhubaneswar vide approval letter no. T/IM-NF/Biochem/21/62 dated 9 Sep 2021 and informed consent was taken from each participant.

### Selection criteria

All patients above 60 years of age with newly diagnosed Hypothyroidism (Clinical or Subclinical) with serum TSH > 5 µIU/mL were included in the study (9). The patients have visited the Endocrinology OPD with complaints like weakness, lethargy, weight gain, sluggishness, lack of interest in work or suffering from constipation etc. The control group consisted of age and sex matched individuals with normal thyroid profile status. Diagnosed cases of diabetes mellitus, hypertension, previous hyperlipidemia, other autoimmune diseases, chronic liver and renal disorders, other endocrine disorders and malnutrition were excluded. Patients on anti-epileptic medication or steroid therapy were also excluded. All participants were asked about their dietary habits and intake of iodized salt in diet was confirmed in all subjects to ensure uniform iodine nutrition status.

The sample size is calculated, keeping the confidence interval as 95% and Power of the study as 80%. To detect a decrease of 10 ml/min/1.73 m^2^ in eGFR among newly diagnosed cases of hypothyroidism compared to control group, a sample size of 64 was the requirement in each group (10). So, total number of subjects recruited were 128 i.e., 64 in each group.

### Data collection procedure

4 ml venous blood was taken in fasting state from the antecubital vein under aseptic conditions in a red-capped plain vacutainer from the subjects. The serum was analysed for thyroid-stimulating hormone (TSH), free T3, free T4, and Anti-TPO using the Siemens Advia Centaur XP chemiluminescence analyser. Renal function tests, Fasting Plasma Glucose (FPG), Postprandial Plasma Glucose (PPG), HbA1C and lipid profile in the blood were conducted using the Beckman Coulter AU5800 autoanalyzer. Quality controls for all the said parameters were satisfactory during the study period. The urinary albumin/creatinine ratio (ACR) was determined and the eGFR was calculated using CKD-EPI Formula (11, 12).

### Statistical tools

Mann-Whitney U test and χ square test were used for tests of statistical significance wherever appropriate. A p value of < 0.05 was considered significant. Statistical analysis was done using SPSS software, Version 26.

## Results

**Figure 1:**
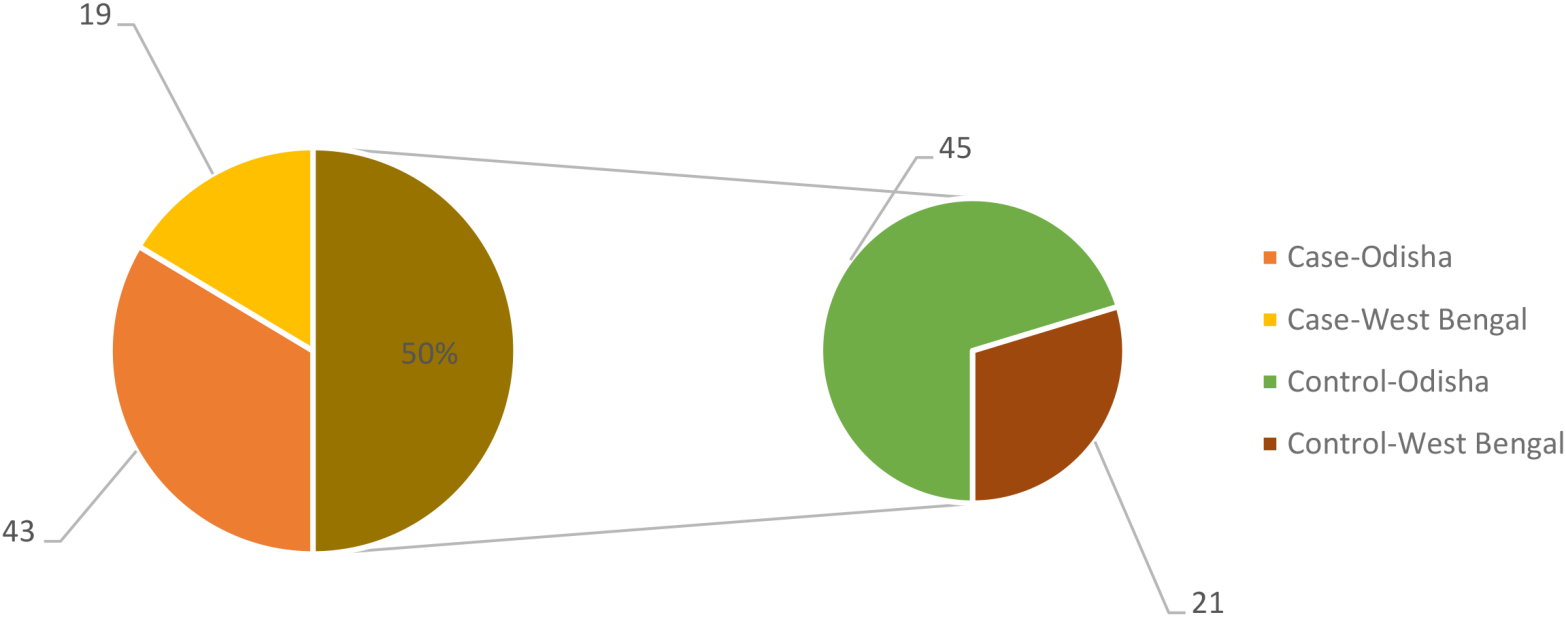
Regional location of participants in case and control group.

The demographic and clinical characteristics of the study groups revealed no significant difference in age and sex distribution. BMI was found to be significantly high among hypothyroid patients compared to control group (**Table 1**). All the participants belonged to the same geographic region of east coast of India, coming from the two provinces of Odisha and West Bengal as depicted in the **Figure 1**. The dietary habit among the participants was also similar as they belong to the similar socioeconomic stratum as prevalent in this geographical area.

**Table 1:** Demographic characteristics of hypothyroid group and healthy controls.

Characteristics	Hypothyroid patients (n=64)	Healthy controls (n=64)	p Value
Age (Years)	67 (63 – 70)	65.5 (63 – 69.75)	0.355
Male	27 (42.2)	35 (54.7)	0.157^χ^
Female	37 (57.8)	29 (45.3)	
Body Mass Index (BMI) (Kg/m^2)^	25.1 (22.5 25.7)	23.45 (21.9 – 24.9)	0.012

^χ^Results represented as Median (quartiles) or Number of Subjects (Percentage) Statistical significance (p value) calculated by Mann-Whitney U test and Chi square test. P value < 0.05 is considered statistically significant.

**Figure 2:**
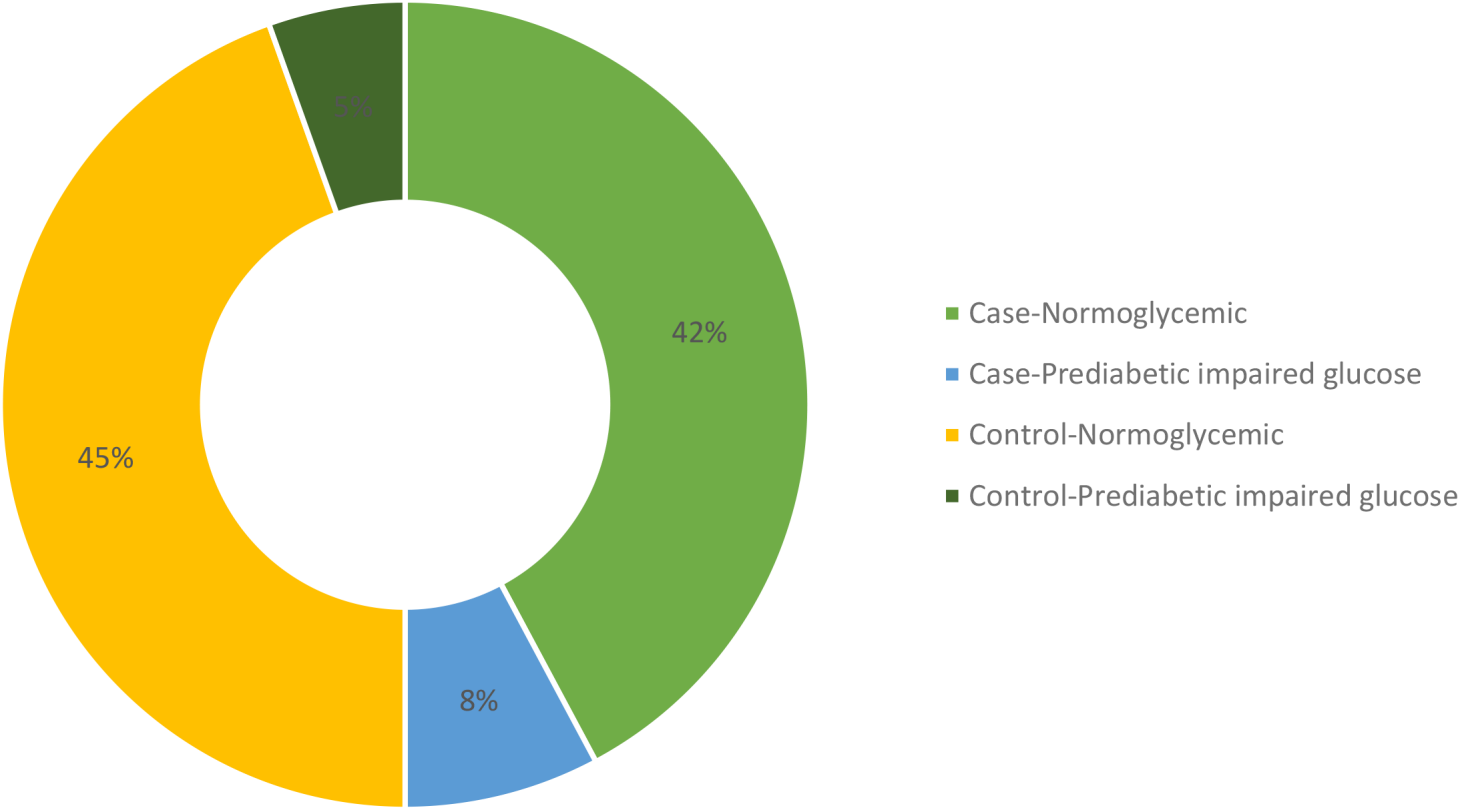
Distribution of glycemic status among participants.

Upon comparing various biochemical parameters between the study groups, it was observed that hypothyroid patients demonstrated significantly elevated TSH levels and decreased levels of free T3 and free T4 in comparison to healthy controls. Furthermore, Anti TPO levels were notably higher in hypothyroid patients than in healthy controls (**Table 2**). On examining renal function and electrolytes across the study groups, it was evident that hypothyroid patients displayed elevated urea levels in comparison to healthy controls, while creatinine levels showed no significant difference between the two cohorts. Moreover, hypothyroid patients exhibited increased uric acid levels relative to healthy controls. Notably, sodium and chloride concentrations were significantly lower in hypothyroid patients, whereas potassium levels remained consistent. Additionally, hypothyroid patients demonstrated a significantly higher urine albumin/creatinine ratio (ACR) compared to healthy controls, indicating potential renal impairment, although eGFR levels were similar between the two groups, suggesting preserved renal function despite alterations in other parameters as shown in **Table 2**. A few participants from both case and control groups were found to be in the prediabetic and impaired glucose tolerance group as evident from their FPG and PPG levels but no confirmed or known case of diabetic was enrolled as all the participants had their HbA1c <6.5%. The distribution of glycemic status among the participants is depicted in **Figure 2**.

**Table 2:** Comparison of biochemical parameters between the study groups.

Parameters	Hypothyroid patients	Healthy controls	p Value
TSH (µIU/ml)	7.035 (6.3 – 10.7)	2.01 (1.53 – 2.87)	<0.001
FT4 (ng/dl)	1.08 (0.71 – 1.23)	1.19 (1.02 – 1.35)	0.003
FT3 (pg/ml)	2.1 (1.26 – 2.7)	2.86 (2.5 – 3.2)	<0.001
Anti TPO (U/ml)	92.25 (50.5 – 207)	16.45 (12.3 – 28)	<0.001
Urea (mg/dL)	30 (21 – 44)	22 (19.25 – 26.75)	0.001
Creatinine (mg/dL)	1.02 (0.89 – 1.28)	0.96 (0.84 – 1.13)	0.176
Uric acid (mg/dL)	5.3 (4.51 – 7)	4.75 (4.51 – 6)	0.024
Sodium (mEq/L)	134 (132 – 136.7)	136 (135 – 139)	<0.001
Potassium (mEq/L)	4.25 ± 0.58	4.29 ± 0.42	0.119
Chloride (mEq/L)	100 (99 – 103.75)	104 (101 – 109)	<0.001
Urine Albumin Creatinine Ratio (ACR) (mg/gram of creatinine)	98.3 (45.2 – 228.5)	22.5 (12.3 – 68.1)	<0.001
eGFR (mL/min/1.73m^2^)	62.5 (45.4 – 79.1)	69.7 (51.6 – 85.2)	0.138
Total Cholesterol (mg/dL)	209.3 (183.5 – 238.8)	184 (135.7 – 222.5)	0.009
Triglyceride (mg/dL)	150 (112.5 – 207)	142 (100.0 – 220.5)	0.922
HDL (mg/dL)	46.3 ± 13	40.78 ± 12.8	0.603
LDL (mg/dL)	139 (118 – 156)	121 (79.75 – 145)	0.002
Fasting Plasma Glucose (mg/dL)	123 (105.5 -152.2)	116 (100.2 – 135)	0.117
Postprandial Plasma Glucose (mg/dL)	166 (137.25 – 222.7)	154 (127.2 – 211.5)	0.081
HbA1C (%)	5.7 (5.1 – 6.07)	5.6 (4.7 – 5.9)	0.187

Data represented as median (quartiles), or mean ± standard deviation wherever applicable. P value <0.05 is considered as statistically significant.

A multiple regression analysis was done to identify the independent predictors of the dependent variable; ACR, reflecting on kidney function. It revealed that among the various parameters, only Anti TPO levels had a significant positive association with ACR, indicating its potential role as an independent predictor of kidney function. Other parameters, including TSH, FT4, FT3, HDL, LDL, total cholesterol, triglycerides, FPG, PPG, HbA1C, and BMI, did not show significant independent effects. These findings suggest that Anti TPO may be a key factor affecting kidney function in the studied population, warranting further investigation and consideration in clinical assessments and interventions (**Table 3**).

**Table 3:** Multiple regression associations of kidney function (ACR) with thyroid function, glycemic status, lipid profile and BMI.

Characteristics	Beta	95% Confidence interval		p Value
		Lower bound	Upper bound	
TSH	0.033	-2.927	3.931	0.772
FT4	-0.018	-120.716	101.381	0.863
FT3	-0.101	-99.434	32.225	0.314
Anti TPO	0.243	0.095	0.925	0.016
HDL	0.232	-0.621	10.694	0.080
LDL	-0.087	-3.651	2.561	0.729
Total Cholesterol	-0.139	-3.106	1.895	0.632
Triglyceride	0.095	-0.366	0.814	0.453
Fasting Plasma Glucose	0.101	-1.424	2.545	0.577
Postprandial Plasma Glucose	-0.146	-2.555	1.090	0.428
HbA1C	0.155	-44.706	131.863	0.330
BMI	0.113	-9.029	38.006	0.225

p value <0.05 is considered statistically significant.

The comparative analysis of renal function between anti TPO positive and anti TPO negative hypothyroid patients revealed notable differences, suggesting a distinct impact of anti TPO positivity on renal health as shown in **Table 4**. Specifically, anti TPO positive patients exhibited significantly higher creatinine and uric acid levels, indicative of impaired renal clearance and altered metabolism. Here, anti-TPO-positive patients had slightly lower urea levels compared to anti-TPO-negative patients which is statistically non-significant. Furthermore, the significantly lower eGFR in the Anti TPO positive group underscores a reduced kidney function compared to their anti TPO negative counterparts. Additionally, the markedly higher urine ACR ratio observed in anti TPO positive patients suggests a greater degree of albuminuria, which is a critical marker of kidney damage. These findings highlight a potential link between Anti TPO antibodies and renal dysfunction in hypothyroid patients, implying that Anti TPO positivity may exacerbate renal impairment.

**Table 4:** Comparison of renal function between Anti-TPO positive and Anti-TPO negative hypothyroid patients.

Parameter	Anti-TPO positive (n=43)	Anti-TPO negative (n=21)	p value
Urea (mg/dL)	30 (20 -44)	33 (22 – 45)	0.327
Creatinine (mg/dL)	1.18 (1 – 1.48)	0.97 (0.88 – 1.19)	0.023
Uric acid (mg/dL)	5.8 (5 – 7.45)	5.1 (4.2 – 6.8)	0.032
Sodium (mEq/L)	134 (131 – 138)	134 (132 – 136)	0.931
Potassium (mEq/L)	4.2 (3.9 – 4.5)	4.4 (4.04 – 4.8)	0.165
Chloride (mEq/L)	100 (99 – 103)	101 (99 – 104)	0.746
eGFR (mL/min/1.73m^2^)	58.6 (34.65 – 77.05)	74.7 (56.8 – 90.1)	0.013
Urine Albumin Creatinine Ratio (ACR) (mg/gram of creatinine)	100.1 (55.07 – 320.25)	52 (30.1 – 141)	0.040

p value <0.05 is considered statistically significant.

## Discussion

The interplay between thyroid dysfunction and renal impairment is particularly significant in the elderly, who are more vulnerable to both conditions. Aging is associated with a decline in renal function and structural changes in the kidney, such as glomerulosclerosis and tubular atrophy, which may exacerbate the effects of hypothyroidism (3). Furthermore, the prevalence of thyroid autoimmunity, including elevated anti-TPO levels, rises with age, making this population particularly relevant for studying the interplay between thyroid autoimmunity and renal health (13). Understanding these associations in the elderly is crucial, as this demographic often presents with overlapping comorbidities, such as cardiovascular disease and diabetes, which are further compounded by thyroid and renal dysfunction. Insights from this study could guide early intervention and management strategies tailored to the elderly.

The demographic characteristics of the study population, including age and sex, showed no significant differences between the study groups. Thyroid hormones are crucial for regulating basal metabolism, thermogenesis, lipid and glucose metabolism, food intake, and fat oxidation. Thyroid dysfunction can lead to alterations in body weight, composition, temperature, and energy expenditure. In particular, hypothyroidism is linked to reduced thermogenesis and metabolic rate, as well as an increased body mass index (BMI) and higher rates of obesity (14). These associations are in line with our study’s finding that hypothyroid patients exhibit higher BMI.

Hypothyroidism is characterized by elevated serum thyroid-stimulating hormone (TSH) levels and decreased circulating free thyroid hormones. Subclinical hypothyroidism (sHT), on the other hand, is defined by normal levels of free thyroid hormones despite elevated TSH (15). In our study also the patient group was observed to have significant elevation in TSH levels and decrease in fT3 and fT4 levels. The destruction of thyroid cells in Hashimoto’s thyroiditis (HT) is linked to several immune processes, both cellular and antibody-mediated, which involve thyroid autoantibodies (TAbs) against thyroid peroxidase (TPO) and thyroglobulin (Tg). Anti-TPO antibodies are more prevalent than anti-Tg antibodies and are more indicative of thyroid disease (16). In our study, we observed that anti-TPO levels were significantly elevated in hypothyroid patients, reinforcing the association between high anti-TPO antibody levels and thyroid dysfunction.

The kidneys are involved in the metabolism and elimination of thyroid hormones and are also target organs for their effects. The renal ramifications of hypothyroidism, encompassing disturbances in water and electrolyte equilibrium and tubular malfunction, are widely acknowledged. However, the alterations in routine biochemical markers within the renal domain in hypothyroidism remain insufficiently elucidated (17). Hypothyroidism induces hemodynamic alterations that result in decreased renal plasma flow and glomerular filtration rate, consequently elevating serum urea and creatinine levels (18). In the present study we noticed a significant increase in serum urea levels in hypothyroid population, while we could not register a significant difference in serum creatinine level among the study groups. Furthermore, increased serum uric acid was observed in hypothyroid patients. Thyroid hormone disturbances can impact purine metabolism, altering uric acid levels and potentially causing hyperuricemia and gout. Hypothyroidism-induced hemodynamic changes can reduce renal plasma flow and glomerular filtration rate, further elevating serum uric acid levels (19).

Thyroid hormones play a pivotal role in maintaining electrolyte homeostasis, exerting multifaceted effects on various ion transporters and channels across different tissues and organs. Sodium and potassium ions constitute essential components of the Na^+^-K^+^ ATPase enzyme, pivotal for cellular membrane function by facilitating the transport of water and nutrients across cell membranes. Thyroid hormones exert regulatory control over sodium-potassium pumps in numerous tissue types (13). In our study we found that serum sodium and chloride levels significantly decrease in patients with hypothyroidism, while potassium has no significant changes, supporting the findings of Bharti et al. (20).

Microalbuminuria serves as a prognostic indicator for diabetic kidney disease (21). Evidence suggests that approximately 10–30% of patients with Hashimoto’s thyroiditis exhibit microproteinuria or nephrotic syndrome (22). This occurrence may be attributed to the release of thyroid antigens, such as thyroglobulin (TG) and thyroperoxidase (TPO), in Hashimoto’s thyroiditis. These antigens, along with their corresponding antibodies (TGAb and TPOAb), form circulating immune complexes that can deposit in the glomerulus, potentially acting as nephritis antigens and contributing to the formation of *in situ* immune complexes (23). In our study we found a significant increase in microalbumin levels in the hypothyroid group, indicating an association of thyroid dysfunction with renal derangement. Meanwhile we did not register a significant difference in eGFR among hypothyroid and normal individuals. To explore further, the hypothyroid group was divided into anti TPO positive and negative subgroups and a comparative analysis was done to see for the effect of anti TPO on the renal system. There was a significant decrease in eGFR this time in anti TPO positive patients along with increased serum creatinine, uric acid, and urine microalbumin levels suggesting a strong association between increased anti TPO levels and renal dysfunction among hypothyroid patients. However, the statistically insignificant lower urea levels in anti-TPO-positive patients compared to anti-TPO-negative patients may be due to the wide variation in blood urea whether due to diurnal pattern, dietary intake, or muscle mass etc.

The involvement of anti-thyroid peroxidase (anti-TPO) antibodies in renal damage could be attributed to the formation of immune complexes. Anti-TPO antibodies, in the presence of thyroid peroxidase (TPO) antigens, may lead to the generation of circulating immune complexes, which deposit in renal tissues. These deposits can trigger an inflammatory response, characterized by complement activation and the recruitment of immune cells, resulting in glomerular injury (22). Additionally, anti-TPO antibodies may disrupt the structural integrity of the glomerular basement membrane, leading to proteinuria and subsequent renal dysfunction. The proinflammatory cytokines released in response to immune complex deposition further exacerbate renal damage, contributing to the progression of chronic kidney disease (CKD) (23). In a study in the urban Middle- Eastern cohort, prevalence of sHT was found to be more among elderly subjects with CKD and older age may explain potentially worse outcomes (24). Wang et al. also stated in their study that study implies that anti-TPO positive status may be useful as a tool to monitor CKD and predict the renal conditions in patients with chronic diseases like type 2 diabetes (25).

In conclusion, our study elucidates a significant association between elevated anti-TPO antibody levels and renal dysfunction among elderly patients with hypothyroidism. We observed that hypothyroid patients with increased anti-TPO levels exhibited a substantial decrease in eGFR, along with elevated serum creatinine, uric acid, and urine microalbumin levels. These findings underscore the potential role of anti-TPO antibodies in contributing to renal impairment in individuals with hypothyroidism. Additionally, in anti-TPO positive young adults, early screening for renal dysfunction may be warranted, as autoimmune processes may lead to progressive renal damage over time. Further research is required to better understand the mechanistic link between anti-TPO antibodies and renal dysfunction and to explore potential therapeutic interventions targeting this pathway.

## Data Availability

The raw data supporting the conclusions of this article will be made available by the authors, without undue reservation.
